# Lyme Carditis Presenting to a Community Hospital in a Non-Endemic Region

**DOI:** 10.7759/cureus.11471

**Published:** 2020-11-13

**Authors:** Patrick Miller, Scott Shinneman

**Affiliations:** 1 Emergency Medicine, Washington State University, Seattle, USA; 2 Emergency Medicine, Overlake Hospital, Bellevue, USA

**Keywords:** lyme, carditis, atrioventricular block, 3rd degree heart block, borreliosis, presyncope, erythema migrans, tick-borne illness, borrelia burgdorferi, non-endemic region

## Abstract

Lyme borreliosis is an infectious disease that is increasing in frequency and can cause various forms of carditis in its disseminated phase. In otherwise healthy patients presenting with new-onset atrio-ventricular dissociation, Lyme carditis must be on the differential; however, due to its rarity in non-endemic regions, the clinician must remain vigilant and keep it on the differential. The objective of this clinical case report is to call attention to the importance of rapid diagnosis of Lyme carditis in regions where the disease is not common. The patient presented in this report is a 27-year-old, previously healthy male complaining of fatigue and presyncope over the past 48 hours who presented to a community ED in western Washington State. He had been traveling the country rock climbing and recalled a febrile illness and rash in the preceding three months. He was found to be in third-degree atrio-ventricular block on admission to the ED and was promptly diagnosed with Lyme carditis. He was hospitalized on telemetry monitoring and was treated with transvenous cardiac pacing and IV ceftriaxone. His atrio-ventricular block gradually resolved and he was discharged without need for permanent pacemaker placement. He was able to return to his active lifestyle of hiking, climbing, and other outdoor recreational activities. This case demonstrates how Lyme carditis must be a foremost consideration in a patient with new-onset conductive heart disease, particularly in patients without risk factors for other causes of atrio-ventricular block. A thorough travel and exposure history must be taken when Lyme carditis is suspected in patients presenting outside of areas where the disease is endemic.

## Introduction

Although Lyme disease is increasing in incidence, and its region of endemicity is expanding, it is sometimes overlooked as a possible diagnosis, particularly in areas where it is less common [1]. The disease can cause severe electrophysiologic and neurological complications and, if left untreated, can lead to death; therefore, prompt diagnosis and treatment is of utmost importance [1-4]. Treatment is relatively benign and most often leads to full resolution without permanent complications [3]. Lyme carditis is a well-described but uncommon complication of early disseminated disease and typically presents with atrio-ventricular blocks, including in patients with no underlying cardiac conductive disease [4].

This case of Lyme carditis that was diagnosed and treated in a community hospital in a non-endemic region. The case is illustrative of a number of the key clinical features of Lyme carditis that will serve as clues to its presence in a patient, including presyncope, previous rash and febrile illness, and history of travel to an endemic region. The patient was a previously healthy individual and had no chronic symptoms preceding the diagnosis of Lyme disease, which suggests that his symptomatology at the time of presentation is almost entirely attributable to the disease. The case also demonstrates the importance of prompt diagnosis and treatment to optimize chances of complete resolution without need for permanent pacemaker placement.

Following the case report is a review of Lyme disease and Lyme carditis.

## Case presentation

Initial presentation

A 27-year-old male presented to a community emergency department in summer of 2016 in western Washington State complaining of lightheadedness and "almost passing out." The patient reported that he had been feeling generally fatigued over the past 48 hours. He was normally very active, hiking and rock climbing frequently, however he felt as though he had not been able to hike at his normal brisk pace. The day before his presentation, while finishing a long hike, he had become lightheaded and nearly lost consciousness, stating that he rapidly lowered himself to the ground. His girlfriend, who accompanied him, confirms that he did not fully lose consciousness. The patient cannot recall any preceding symptoms; he specifically denies having chest pain, palpitations, shortness of breath, headache, or any focal neurological deficits. Several seconds after the episode, the patient felt completely back to his recent baseline. He had no persistent lightheadedness.

Upon returning from the hike that day, the patient visited a local urgent care. He had no symptoms at the time and was noted to have dry mucus membranes on physical exam. His work-up there included complete blood count, comprehensive metabolic panel, and electrocardiogram, which were normal. The patient was diagnosed with dehydration and presyncope and was discharged to home.

At home, the patient rehydrated orally and went to bed. He awoke at 02:00 with recurrent symptoms so he drove to the emergency department.

Upon arrival, the patient was placed on a monitor in the triage room and was noted by the triage nurse to be in complete atrioventricular dissociation. During triage, the patient briefly lost consciousness, during which time the triage nurse noted an eight-second ventricular pause on the cardiac monitor. External pacer/defibrillator pads were placed and the patient was brought back to a trauma room. On initial physician interview, the patient noted continued lightheadedness but denied chest pain, palpitations, jaw or arm pain, nausea, edema, or shortness of breath. He also denied headache, focal weakness, and speech or coordination changes. He reported no fever or respiratory symptoms in the past week.

The patient had no past medical or surgical history. He received all routine pediatric vaccinations and screening studies. He was not prescribed any medications and denied using any supplements or alternative medical treatments. He had no known drug allergies. He had a family history of hypertension in his mother and coronary artery disease in his father, diagnosed in his 60s. There was also a family history of colorectal cancer in his father. He denied any known family history of sudden cardiac death or cardiac arrhythmias, although he admitted to not being well informed about his family history. The patient was a non-smoker, drank one to two alcoholic drinks per week, and denied recreational or intravenous drug use.

During the travel history, the patient did report having traveled extensively throughout the United States over the past year to participate in outdoor recreational activities. He recalled camping in the Northeast region two months ago, after which he had some low grade fever and a red rash on his right shoulder. He described the rash as circular and gradually enlarging. He did not recall any central clearing. The fever and rash spontaneously resolved and he did not seek medical attention for these. He had no further symptoms after that, up until 48 hours prior to his presentation to the emergency department.

A complete review of systems was obtained and was negative. Vital signs were blood pressure: 112/65; heart rate: 62; respiratory rate: 12; temperature: 98.5.

Physical exam revealed a fit-appearing young male who was mildly diaphoretic but was alert and fully oriented and in no acute medical distress. Cardiac exam demonstrated a borderline bradycardic rate. There were no murmurs, rubs, or gallops. There was no extremity edema and peripheral pulses were symmetric. There was no jugular venous distention. Breath sounds were clear to auscultation and symmetric. His abdomen was soft and non-tender. The remainder of the physical examination was without noted abnormalities. Notably, there was no rash, focal weakness, or joint tenderness or effusion. He had a normal neurologic exam.

**Figure 1:**
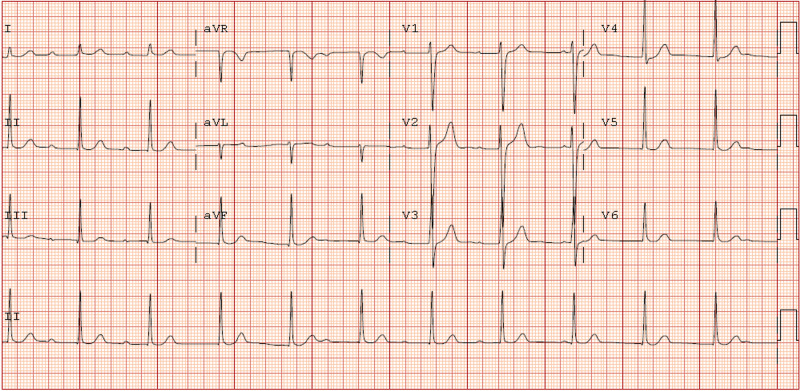
Twelve lead ECG taken shortly after the patient's initial presentation to the emergency department demonstrating atrioventricular (AV) dissociation. There are no other ECG abnormalities.

An electrocardiogram was obtained rapidly after the patient’s admission to the emergency department. This showed atrioventricular (AV) dissociation with accelerated junctional rhythm. Axis was normal. There were no acute ST elevation or depression and no T wave abnormalities. See Figure 1 for the ECG.

Complete blood count in the emergency department showed a white blood cell count of 8,500 per microliter (reference range 3,800-11,000 per microliter), hematocrit of 41.2% (reference range 39-50%), and a platelet count of 248,000 per microliter (150,000-400,000 per microliter). White blood cell differential was within the hospital laboratory’s established normal limits.

A comprehensive metabolic panel revealed a glucose of 97 mg/dL (reference range 65-99 mg/dL), creatinine of 0.80 mg/dL (reference range 0.7-1.30 mg/dL), blood urea nitrogen (BUN) of 15 mg/dL (reference range 7-18 mg/dL), sodium of 140 mmol/L (reference range 136-145 mmol/L), and potassium of 3.6 mmol/L (reference range 3.5-5.1). Transaminases and alkaline phosphatase were within normal laboratory reference ranges.

Urine toxicology screen was negative for any drugs of abuse.

A point-of-care troponin was not elevated outside of the reference range. A repeat troponin was checked 4.5 hours later and was also within normal limits. 

Subsequent serial ECGs demonstrated periods of junctional rhythm slower than atrial rhythm, confirming third-degree heart block.

**Table 1:** Differential diagnosis for third degree atrioventricular block [1-7] HOCM: Hypertrophic obstructive cardiomyopathy

Differential diagnosis for third degree atrioventricular block includes:
Primary conductive heart disease
Acute coronary syndrome, especially involving the right coronary artery
Ischemic cardiomyopathy
Infiltrative cardiomyopathy such as hemochromatosis, sarcoidosis, or amyloidosis
Infective endocarditis and endocardial abscess
Medication effect, including beta adrenergic antagonists, calcium channel blockers, and digoxin
Metabolic derangement including hyperkalemia and azotemia
Rheumatic/autoimmune diseases, such as acute rheumatic fever, systemic lupus erythematosus, sarcoidosis, or systemic sclerosis
Lyme carditis or other infectious carditis such as Chagas disease, diphtheria infection, or tertiary syphilis
Congenital and genetic heart diseases including HOCM
Malignancy
Elevated vagal tone
Iatrogenic, such as failure of implanted pacemaker or complication from ablation procedure

Intervention

The patient was given a 1-liter bolus of intravenous normal saline and was admitted to a cardiac telemetry floor. A transvenous pacemaker was placed. Blood was sent for serologic testing for *Borrelia burgdorferi* antibodies. He was started on empiric therapy with 2 grams of IV ceftriaxone given once daily on the presumption of Lyme carditis as well as maintenance IV normal saline.

Serial laboratory evaluation each morning consisted of a complete blood count and comprehensive metabolic panel. These laboratory studies did not fall significantly outside of reference ranges throughout the course of the patient’s hospitalization. Serial electrogardiograms were obtained and showed persistent complete atrioventricular block in the first three days of his hospitalization. Additional diagnostic studies that were ordered during the patient’s hospital stay included an echocardiogram, which revealed a normal left ventricular wall thickness, normal left ventricular ejection fraction of 55-60% (reference range 55-60%), and no significant valvular regurgitation or stenosis. There was no evidence of diastolic dysfunction to suggest infiltrative cardiomyopathy. There was no pericardial effusion. A thyroid stimulating hormone level was checked and was 1.210 microIU/mL (reference range 0.358- 3.740 microIU/mL).

Outcomes

The patient’s enzyme-linked immunosorbent assay (ELISA) and subsequent Western blot serology testing returned positive for *Borrelia burgdorferi* antibodies, suggesting active infection. He remained in the hospital for 10 days during which time his cardiac telemetry monitoring showed gradually lengthening intervals of Mobitz I second-degree heart block. Following seven days of treatment, there were no further intervals of third-degree heart block. Upon discharge from the hospital, pacemaker wire was removed. The patient followed up with a family medicine physician one week following his discharge. ECG done at that time showed normal sinus rhythm.

## Discussion

Etiology/Pathogenesis

Lyme disease (or Lyme borelliosis) is caused by infection with *Borrelia burgdorferi*, a poorly Gram-staining, Giemsa stain-positive spirochete bacterium. It is transmitted by the bite of the Ixodes family of ticks [1]. The clinical incubation period for the bacterium is between three and 32 days [1]. The bacterium most often causes a localized infection at the site of the bite of an infected tick; upon injection by the tick and initial recognition of the bacterium by innate and adaptive immune elements, a local inflammatory reaction occurs, causing the stereotypical rash of erythema migrans [1,2].

In the ensuing days and weeks, the bacterium is able to disseminate very broadly through adherence to a wide variety of cell-surface glycoproteins and matrix glycosaminoglycans [1]. This process appears to be promoted through binding to fibrinogen and related coagulation cascade mediators [1]. Additionally, the bacterium undergoes a number of antigenic variations and downregulations which help it to evade immune detection [1,3].

Although bacteria have been detected in infected individuals in the muscle, spleen, meninges, and elsewhere, its infection of the myocardium and nerves and nerve sheaths that produce the hallmark symptoms of the early disseminated phase. Infected tissues develop inflammation and vasculitis due to immune cell extravasation and cytokine release [1].

Humans are a dead-end host for *B. burgdorferi*. Over the course of months to years, the bacterial burden is gradually reduced by immune response [1]. During this time, persistent localized areas of infection can cause the ongoing symptoms of the late-disseminated stage of the disease. The bacteria are eventually completely immunologically eradicated.

Epidemiology

There are over 30,000 cases of Lyme disease reported annually in the United States [1]. The disease has a distinct geographic pattern, with the majority of cases occurring in the Northeastern United States [3,5]. The disease is also present in the Midwest and in Northern California and Oregon [3,5]. The disease has increased in both incidence and in geographical distribution over the past several decades [1,3].

Clinical presentation and evaluation

The clinical presentation of Lyme borreliosis can be divided into 3 stages: local (stage 1), early disseminated (stage 2), and late disseminated (stage 3).

During the local stage, within the several days following a known or unnoticed tick bite, an erythematous rash known as erythema migrans develops [1-4]. This most often occurs at the site of the tick bite. The rash is erythematous, flat, and is not typically pruritic or painful [2]. It gradually increases in size and may develop central clearing or target-like rings of erythema [1-4]. The early stage can also present with fever, fatigue, and arthralgias, but other symptoms are uncommon [1-4]. The erythema migrans rash can have a characteristic appearance that would clue a clinician into the diagnosis, particularly in an endemic region. However, the rash resolves spontaneously and many patients do not seek medical care for it.

The early disseminated stage occurs within the several weeks following the development of the initial erythema migrans lesion and is marked by systemic symptoms including malaise and fatigue and is often accompanied by further dermatologic manifestations such as erythematous, annular skin lesions in a diffuse distribution [1,2]. Although the early disseminated phase can affect nearly every organ system, especially the brain and meninges, the classical manifestations are atrioventricular heart block and cranial nerve VII palsy which can be bilateral [1,2,5].

Estimations of the occurrence of Lyme carditis in cases of Lyme disease fall between 0.3% and 4% [3]. Lyme carditis, like other manifestations of Lyme borreliosis, occurs due to acute inflammation and immune response due to direct invasion of pathogens [1,3]. It affects men three times more often than women and most typically presents with complete atrioventricular dissociation, however this is most often temporary given prompt treatment [3]. It is reported to occur more frequently in young, otherwise healthy male patients [5]. Permanent pacemaker placement is not indicated, particularly in young and otherwise healthy individuals [3,7]. As of 2014, there had been seven reported cases of death due to Lyme carditis [5].

Within several months following early disseminated infection, most patients enter the third stage of Lyme borreliosis, which is the late disseminated phase [1,3]. This is marked by improvement in neurologic and constitutional symptoms but ongoing migratory polyarthritis that may take years to resolve.

Although most clinicians who practice in regions where Lyme borreliosis is endemic will be well aware of its presentation and will have a high baseline index of suspicion, it is often overlooked in non-endemic regions, such as the Pacific Northwest region in this case. Any patient with rash typical of Lyme borreliosis, new atrioventricular heart block, cranial nerve palsies, and migratory polyarthritis should be questioned about travel history and exposures to ticks.

Treatment

Treatment of Lyme borreliosis is dependent on the manifestations of the disease. Adults with early (localized) infection can be treated with a 14-21 day course of doxycycline, 100 mg [1,2,5]. Alternative therapy, or first-line therapy in children, is a 14-21 day course of amoxicillin [1]. For cases with any evidence of meningitis, encephalitis, or carditis, 2 grams of IV ceftriaxone should be given once daily for 14-28 days [1,3]. Once patients are stabilized and cardiac or neurologic involvement is resolved, treatment can be switched to oral antibiotics [1,2].

For patients with high-grade atrioventricular block, external pacemaker-defibrillator pads should be placed and a temporary pacemaker should be inserted after hospital admission [7]. Any patient with meningitis, encephalitis, or carditis should be admitted under telemetry monitoring and given supportive care.

Prognosis and complications

When left untreated, Lyme borreliosis can be life-threatening [5]. Major causes of morbidity and mortality include cardiac arrest due to high-grade atrioventricular block, severe meningitis with elevated increased intracranial pressure (ICP), and severe sepsis or septic shock [1,3]. Nonetheless, Lyme borreliosis is self-limiting over the course of months to years in the majority of cases [1].

Patients who are treated early on in the course of the disease typically recover without complication or further symptoms. Patients with cardiac involvement who are treated most often recover fully and do not require permanent pacemaker placement [5].

Some patients experience a post-infectious syndrome to include migratory arthritis, chronic malaise and fatigue, and neurocognitive impairment, among others [1,3]. 

## Conclusions

Although Lyme carditis is a relatively common complication of borreliosis, it is often unsuspected when it presents in non-endemic regions. Any patient with a high-grade heart block should be thoroughly interviewed about travel and exposure history, in particular when there is no alternative explanation for AV block. While third-degree atrioventricular heart block can be life-threatening if left untreated, prompt intervention with transvenous pacing and definitive treatment with IV antibiotics typically prevents the need for permanent pacemaker placement in patients with Lyme carditis.
